# Immunoanalysis of different antigenic preparations of *Angiostrongylus cantonensis* for neuroangiostrongyliasis diagnosis improvement

**DOI:** 10.1590/0074-02760220086

**Published:** 2022-10-03

**Authors:** Leyva Cecília Vieira de Melo, Felipe Corrêa Rezende de Souza, Amanda de Oliveira Baccin, Dan Jessé Gonçalves da Mota, Vera Lucia Pereira-Chioccola, Pedro Luiz Silva Pinto

**Affiliations:** 1 Instituto Adolfo Lutz, Centro de Parasitologia e Micologia, Núcleo de Enteroparasitas, São Paulo, SP, Brasil; 2 Secretaria Estadual da Saúde de São Paulo, Coordenadoria do Controle de Doenças, Programa de Pós-Graduação, São Paulo, SP, Brasil; 3 Unidade de Vigilância em Saúde, São Paulo, SP, Brasil; 4 Instituto Adolfo Lutz, Centro de Parasitologia e Micologia, Laboratório de Biologia Molecular de Parasitas e Fungos, São Paulo, SP, Brasil

**Keywords:** *Angiostrongylus* infection, Angiostrongylus cantonensis, meningitis, immunodiagnosis, eosinophilia

## Abstract

**BACKGROUND:**

*Angiostrongylus cantonensis* is the etiological agent of neuroangiostrongyliasis in humans, which is developed in gastropods and vertebrate species, mainly rodents. Human transmission occurs through consumption of molluscs and paratenic hosts infected with L3, and the migration of larvae to the central nervous system causes eosinophilic meningitis. Laboratory diagnosis is based on molecular and immunological tests, using young or adult females as a source of antigens. However, these tests give positive results only after several weeks of symptoms onset and also cross-reactions with others parasite infections may occur.

**OBJECTIVES:**

The purpose of this work was to study different antigenic preparations of distinct evolutionary phases of *A. cantonensis*, in order to improve serological techniques for disease immunodiagnosis.

**METHODS:**

For this purpose, antigenic fractions of different evolutionary forms were evaluated by Dot-enzyme-linked immunosorbent assay (Dot-ELISA) and Western blot using serum samples.

**FINDINGS:**

All analysed fractions showed reactivity with serum samples from patients with neuroangiostrongyliasis, especially female membrane alkaline (FAM) and female soluble alkaline (FAS) fractions together with female soluble saline (FSS), improving the technique specificity.

**MAIN CONCLUSIONS:**

The results point to the possibility of use of raw female antigens in association with alkaline membrane antigens extracted from adult worms to aid in diagnosis and helps initiate neuroangiostrongyliasis surveillance and control actions.

*Angiostrongylus cantonensis* is an etiologic agent of neuroangiostrongyliasis, main pathology associated with eosinophilic meningitis, which has been causing concern to public health agencies in several countries due to its emerging pattern. The metastrongilids were described by Chen in 1933,^1^ from specimens of *Rattus norvegicus* captured in a Chinese city of Canton (now Guangzhou). The first record in humans occurred in 1945^2^ and data point to 2,877 human cases worldwide,^3^ although the data are non-updated and probably underestimated. Angiostrongyliasis is endemic in Pacific Islands, Southeast Asia and China.^4^ ^,^ ^5^ The first records in the Americas occurred in Cuba, Costa Rica, Jamaica and Brazil.^6^ ^,^ ^7^ ^,^ ^8^

The parasite evolutionary cycle involves several snails and slugs species as intermediate hosts and some vertebrates, mainly rodents, as definitive hosts.^9^ ^,^ ^10^ Reptiles and some invertebrates may participate as paratenic hosts.^11^

The human infection occurs accidentally with the ingestion of infected molluscs or paratenic hosts, in nature or undercooked. However, *A. cantonensis* does not complete its cycle in humans and L3 larvae migration and death in central nervous system (CNS) causes eosinophilic meningitis or meningoencephalitis, which are induced by intense reactivity to parasitic components.^12^ In this sense, cultural factors play an important role in disease transmission.^6^ ^,^ ^5^

Clinical symptoms are consistent with typical meningitis,^13^ such as: headache, photophobia, neck stiffness, fatigue, hyperesthesia, vomiting, paresthesia, sensory and motor disturbances, leg pain, lack of reflexes, bowel and bladder disorders, and labial hypertension. In more severe cases tetraparesis, coma, and death may occur.^14^ ^,^ ^15^ Another consequence is an increase in blood eosinophils (> 5%) and/or in cerebrospinal fluid (CSF) (> 10%).

There is no specific treatment for neuroangiostrongyliasis, as there is no consensus among researchers on administration of anthelmintic.^12^ Recent research suggests that administration of mebendazole may be beneficial, but its use is conditioned to the infection stage and the severity of symptoms.^16^ Thus, corticosteroid therapies remain the best approach for treatment.^4^ ^,^ ^16^

Regarding immunodiagnosis, several techniques have already been developed, but the enzyme-linked immunosorbent assay (ELISA) and Western blot are most used in countries that adopt this kind of diagnosis to help clarify cases. However, sensitivity and specificity of those tests vary depending on characteristics of antigens used in reactions.^17^ ^,^ ^18^ Studies pointed out that low molecular weight antigens (29 kDa and 31 kDa) identified in different evolutionary parasite stages are considered as serological markers of greater efficiency in *A. cantonensis* infections.^18^ ^,^ ^19^ Unfortunately, the occurrence of cross-reactions with other helminthiasis still represents a limiting factor in the interpretation of results.^12^ In addition, the absence of specific antibodies in the acute phase, also, compromises effectiveness of immunological methods in early diagnosis of neuroangiostrongyliasis.^17^ Therefore, methodologies recently used for confirmatory diagnosis of *A. cantonensis* infection in humans are the direct microscopy observation of larvae in CSF or real time polymerase chain reaction (qPCR) for parasite DNA research.^4^ ^,^ ^12^ In both methods, the positivity depends on parasite load^18^ and infection stage.^20^ In qPCR, the detection of parasite DNA may vary according to infection course, intercalating between positive and negative results for the same patient.^21^ Next-generation sequencing is currently studied for diagnosis of eosinophilic meningitis, enabling detection of several pathogens, including *A. cantonensis*.^22^ ^,^ ^23^ However, this methodology is not yet available for implementation in diagnostic routines.

Therefore, the diagnosis, even in endemic areas, is based on clinical evolution, an increase of eosinophils in blood and/or CSF and reactivity of serological tests. In some cases, the evidence of consumption or contact of patients with intermediate or paratenic hosts.^16^ ^,^ ^17^ Brazil is one of the countries that consider serological results for neuroangiostrongyliasis diagnosis, however, with parsimony, observing symptoms and epidemiological aspects, which are usually discussed with medical teams and surveillance agencies. The tests applied are ELISA or Dot-ELISA for screening, and Western blot as a confirmatory test.^8^ ^,^ ^24^ In addition, qPCR^7^ and CSF larvae direct microscopic observation are adopted in suspected cases, as well as environmental investigation once a case is confirmed.

Given the above, it is clear that advances in research do not seem to follow the emerging feature of this infection, as immunological tests capable of discriminating it from other helminthiasis or even detecting infection at the onset of symptoms have not yet been developed. Thus, the aim of this study was to evaluate different antigenic preparations from distinct evolutionary phases of *A. cantonensis*, as well as, from the reproductive system of adult females, in order to contribute to improvement of immunological techniques for diagnosis.

## MATERIALS AND METHODS

*Ethics statement* - The present study was approved by the Scientific Technical Council (CTC) n. 68I/2016 and by the Research Ethics Committee (CEP) n. 2.271.089/2017. As for the use of animals, it is part of a project submitted to the Ethics Committee on use of Animals (CEUA/PASTEUR) n. 04/2012 with title: Ecoepidemiology and Laboratory Diagnosis of Angiostrongyliasis in State of São Paulo.

*Clinical samples* - This is a retrospective study, which tested 112 serum samples received and stored at the Enteroparasite Nucleus, Adolfo Lutz Institute (IAL), of which, 46 sera were from patients with eosinophilic meningitis that were divided in 17 with confirmed neuroangiostrongyliasis, 25 unconfirmed and four with indeterminate results. The other 46 sera were from patients with other helminthiasis (strongyloidiasis, schistosomiasis, ascariasis, toxocariasis and neurocysticercosis) and 20 sera were from patients without helminthiasis. Characterisation of reactive and non-reactive samples for angiostrongyliasis was based on tests performed with soluble saline antigens from adult females. The sera tested in this study were sent to the collaborating center “Pontifical Catholic University of Rio Grande do Sul, Brazil”, which was responsible for the laboratory diagnosis of angiostrongyliasis in Brazil, that include ELISA and Western blot using heterologous antigen of young female worm.^25^

*Obtainment of larvae and worms of A. cantonensis* - Specimens were obtained from the cycle of *A. cantonensis*, strain Village,^26^ maintained by Enteroparasite Nucleus of IAL, in which groups of 15 snails of *Biomphalaria glabrata* species (BH strain) and three Wistar rats provided by the Institute of Tropical Medicine (IMT) were used. The procedures adopted for maintenance of parasite consisted of infection of molluscs with approximately 700 L1, via opening of shells, based on modifications of methodology adopted by Yousif and Lämmler.^27^ Rats were infected by inoculation via esophageal tube containing 30 L3, following methodology adapted from Wallace and Rosen.^28^

**Fig. 1:**
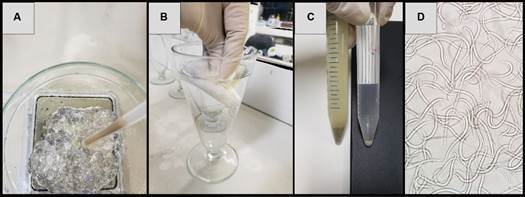
purification of *Angiostrongylus cantonensis* L1 larvae with collagen. A: emulsification of collagen with the 1st Rugai sediment; B: 2nd Rugai in cold dechlorinated water; C: comparison between the sediments of the 1st (left) and 2nd Rugai (right); D: purified L1 larvae.

*Extraction of L1 and L3 from A. cantonensis* - Rugai method^29^ was used for acquisition of 500 mg of L1 from feces of rodents with 45 days of infection and larvae were purified with semi-hydrolysed collagen. As shown in Fig. 1, sediment was mixed with 10 g of collagen with a density between 1375 and 1475 and submitted again to the Rugai method using water at room temperature. Purified L1 larvae were collected and frozen at -20ºC. For acquisition of 500 mg of L3, infected molluscs were crushed and submitted to Rugai method using pepsin 4%, HCl 0.7% in dechlorinated water.

**Fig. 2:**
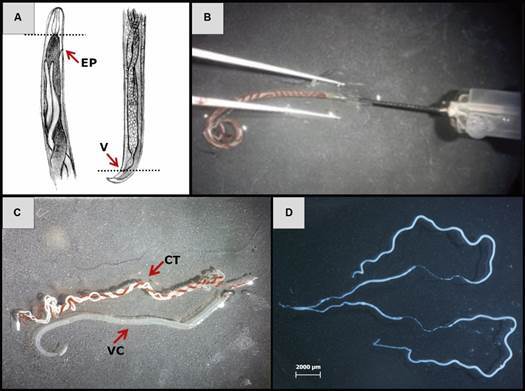
extraction of the reproductive system of adult females of *Angiostrongylus cantonensis*. A: identification of the sites for the sections (PE: excretory pore; TO: ovary terminations; V: vulva; An: anus); B: insertion of the needle into the anterior part of the worm; C: female after viscera extraction (CT = cuticle, and CV = visceral content); D: extracted female reproductive system.

*Collection of worms and removal of reproductive tract of adult females of A. cantonensis* - Adult worms were removed from pulmonary arteries from rodents after 45 days of infection. For such, rodents were euthanised and submitted to perfusion of their circulatory systems. In addition, transverse cuts were performed in anterior and posterior regions of the worm to obtain the reproductive tract of females. Subsequently, an insulin needle was introduced through the anterior cavity of worms, while cuticle was guided along the needle with the aid of a tweezer. Thus, internal organs were expelled through the posterior cavity and the reproductive system was separated from the intestine and frozen at -20ºC (Fig. 2).

*Obtainment of soluble and membrane antigens* - L1 larvae and adult worms (male and female) of *A. cantonensis* were used for extraction of soluble saline (SS), membrane saline (SM), soluble alkaline (AS) and membrane alkaline (AM) antigenic preparations. However, due to limited L3 and female reproductive tract material available, only SS and SM antigens were extracted. Thus, 16 preparations were investigated: female soluble saline (FSS) and membrane saline (FSM); female soluble alkaline (FAS) and membrane alkaline (FAM); male soluble saline (MSS) and membrane saline (MSM); male soluble alkaline (MAS) and membrane alkaline (MAM); L1 soluble saline (L1SS) and membrane saline (L1SM); L1 soluble alkaline (L1AS) and membrane alkaline (L1AM); L3 soluble saline (L3SS) and membrane saline (L3SM); and uterus soluble saline (UTSS) and membrane saline (UTSM).

Soluble antigens were obtained according to modifications of technique described by Machado et al.^30^ using PBS 0.01M, pH 7.2 and protease inhibitor cocktail (Sigma^®^ P2714). For that, liquid nitrogen was used, interspersing between freezing and thawing in a water bath at 37ºC for ten times and including macerations with a pestle in the intervals. Subsequently, samples were fragmented with the aid of an ultra-homogeniser (Ultra Stirrer-MecLab^®^) in ten one-minute cycles, with two minutes of rest on ice after each cycle. Next step consisted of centrifugation of samples at 9,600xg at 4ºC for 30 min and the supernatant was frozen at -20ºC. For extraction of membrane antigens, sodium dodecyl sulfate (SDS) 10% was added to pellets. Then, samples were heated for 5 min at 100ºC in a dry bath (AccuBlock™ - Labnet^®^), centrifuged and stored as described above. The same procedure was adopted to obtain alkaline fractions, replacing NaOH 0.15M in PBS, 0.01M. Next, samples were pH balanced with HCl before being dialysed overnight against PBS 0.01M, pH 7.2. Following this period, samples were centrifuged and stored. Protein concentrations were determined by Bradford method,^31^ using bovine serum albumin (BSA) as standard.

*Analysis of protein profiles of antigenic fractions* - Protein profiles were determined by SDS-PAGE electrophoretic analysis, according to a methodology adapted from Laemmli,^32^ using acrylamide gel for stacking at 5% and for separation with a gradient from 10% to 18%. The conditions used were 30V, 500mA and 250W, changing to 90V after stacking until end of run and gels were stained with Coomassie Blue R250.

To perform Dot-ELISA, a nitrocellulose membrane with 0.45µm porosity and protein concentration of 1.25 µg was used. Dilutions of sera, CSF and peroxidase-labeled anti-human IgG conjugate were 1:160, 1:2 and 1:4000, respectively. To perform Western blot, strips of nitrocellulose membrane (0.45 µm) were made, after transferring electrophoresis products using the TransBlot^®^ SD Semi-dry Transfer Cell device at 20V, 400mA and 250W, for 90 min. The antigen concentrations and serum dilutions which were used followed the same criteria as for Dot-ELISA.

*Data analysis* - Results were analysed determining sensitivity, specificity, accuracy, positive and negative predictive values. In addition, Kappa (K) coefficient^33^ was calculated for agreement analysis using IBM^®^ SPSS software and following classification according Viera and Garrett:^34^ was used: 0.81 to 1.00 = almost perfect; 0.61 to 0.80 = substantial (strong); 0.41 to 0.60 = moderate; 0.21 to 0.40 = reasonable; 0.00 to 0.20 = weak; and < 0.00 = insignificant.

## RESULTS

**Fig. 3:**
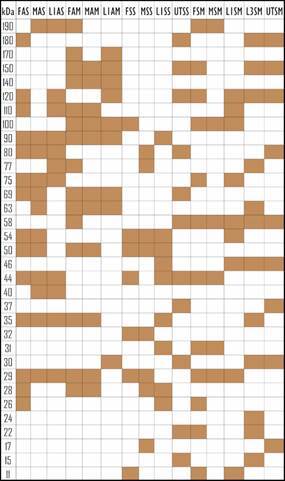
scheme of protein fractions of extracts obtained from *Angiostrongylus cantonensis*. FAS: female soluble alkaline; MAS: male soluble alkaline; L1AS: L1 soluble alkaline; FAM: female membrane alkaline; MAM: male membrane alkaline; L1AM: L1 membrane alkaline; FSS: female soluble saline; MSS: male soluble saline; L1SS: L1 soluble saline; FSM: female membrane saline; MSM: male membrane saline; L1SM: L1 membrane saline; L3SM: L3 membrane saline; UTSS: soluble saline of uterus and ovaries; UTSM: Saline membrane of uterus and ovaries.

Protein fractions with molecular masses between 190 kDa and 11 kDa were identified in the studied samples. The antigens that presented the highest number of visible bands were FAM, FAS, MAS, FMS and L3SM, while extracts with lower amounts of protein fractions were MSM and FSS (Fig. 3). As for bands shared by different extracts, the 29 kDa band was not visible only in L1AM, L1SS, UTSS, UTSM and L3SM. On the other hand, the 24 kDa band was expressed only by L3SM.

**Fig. 4:**
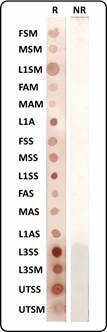
examples of Dot-enzyme-linked immunosorbent assay (ELISA) results on reactive (R) and non-reactive (NR) samples.

In Dot-ELISA, using FSS fraction as reference antigen, L3SS fraction was closest to FSS, showing reagent results for 16 of 17 serum samples from patients with a confirmed diagnosis of neuroangiostrongyliasis, followed by MSS, L1AS, L3SM and UTSS (14/17). On the other hand, FAM and MAM were the ones that least identified antibodies (6/17). Fig. 4 shows examples of reactions obtained by Dot-ELISA.

Regarding sensitivity, L3SS was the antigen that presented the highest value (94.12%) compared to 100% of FSS, followed by MSS, L1AS, L3SM and UTSS, with 82.35% each. However, specificity was higher in FAM (91.21%), UTSM (90.91%) and MAM (89.01%), which suggests that membrane antigens are more sensitive for detection of anti-*A. cantonensis* IgG antibodies, especially alkaline ones (Table I).

On the other hand, the possibility of a reagent producing a real result, determined by positive predictive value (PPV), had the best result for L3SM, with 53.85% compared to FSS, which presented 38.64%. In addition, negative predictive values (NPVs) of all extracts were above 85%, and L3SS was closest to FSS, with 97.83%. However, the highest accuracy value was observed for FAS, with 83.33%. Other antigens remained between 52.78% (L1SS) and 82.41% (MSM) (Table I).

Regarding the concordance analysis of Dot-ELISA results in sera from patients with and without neuroangiostrongyliasis or other helminthiasis, none of antigenic fractions showed ALMOST PERFECT or SUBSTANTIAL agreement with antigen already standardised for disease diagnosis (FSS). However, L1SM, L1AS, L1AM, MSS, FAM and MAM antigens showed a REASONABLE concordance rating, while FSM and L1SM were the ones that had least concordance with FSS, considered WEAK (Table II).

**TABLE I:** Sensitivity, specificity, positive predictive value (PPV), negative predictive value (NPV) and accuracy of the Dot-enzyme-linked immunosorbent assay (ELISA) technique, comparing between female soluble saline (FSS) and the other antigens addressed in this work

Antigen	n	Sensivity (%)	Specificity (%)	PPV (%)	NPV (%)	Accuracy (%)
FSS	108	100,00	70,33	38,64	100,00	75,00
FSM	108	70,59	82,42	42,86	93,75	80,56
MSM	108	76,47	83,52	46,43	95,00	82,41
L1SM	108	58,82	83,52	40,00	91,57	79,63
FAM	108	35,29	91,21	42,86	88,30	82,41
MAM	108	35,29	89,01	37,50	88,04	80,56
L1AM	108	76,47	71,43	33,33	94,20	72,22
MSS	108	82,35	65,93	31,11	95,24	68,52
L1SS	108	76,47	48,35	21,67	91,67	52,78
FAS	108	76,47	84,62	48,15	95,06	83,33
MAS	108	70,59	82,42	42,86	93,75	80,56
L1AS	108	82,35	70,33	34,15	95,52	72,22
L3SS	84	94,12	67,16	42,11	97,83	72,62
L3SM	84	82,35	82,09	53,85	94,83	82,14
UTSS	88	82,35	67,61	37,84	94,12	70,45
UTSM	83	41,18	90,91	53,85	85,71	80,72

**TABLE II:** Analysis of agreement by the Kappa test of the Dot-enzyme-linked immunosorbent assay (ELISA) results in serum samples against the antigenic fractions studied, compared with female soluble saline (FSS)

Antigen	Kappa	Classification	p
FAS	0,493	MODERATE	0,000
MSM	0,475	MODERATE	0,000
MAS	0,420	MODERATE	0,000
L1SM	0,355	REASONABLE	0,000
L1AS	0,335	REASONABLE	0,000
L1AM	0,326	REASONABLE	0,000
MSS	0,289	REASONABLE	0,000
FAM	0,286	REASONABLE	0,003
MAM	0,249	REASONABLE	0,010
L1SS	0,122	WEAK	0,059
FSM	0,001	WEAK	0,983
L3SS^*a*^	-	-	-
L3SM^*a*^	-	-	-
UTSS^*a*^	-	-	-
UTSM^*a*^	-	-	-

*a*: antigens not analysed, for did not contemplatenon-reactive samples for neuroangiostrongyliasis or other helminths.

In this case, almost perfect or substantial agreement between FSS fraction and other antigens is not objective, as there would be no change and consequent improvement in diagnosis. Thus, when results of accuracy and K values were considered together, antigens with moderate agreement were MSM, FAS and MAS, of which FAS presented the highest specificity index (84.62%). Among reasonable ones, those that presented best specificity indices were FAM (91.21%) and MAM (89.01%), and all of these antigens presented PPVs close to each other, exceeding values found for FSS. Another antigenic fraction with higher values of specificity, PPV and NPV than FSS was UTSS, with 90.91%, 53.85% and 85.71% respectively. Concordance index was not calculated because it was not possible to perform tests in patients without studied disease or other helminthiasis, due to scarcity of these antigenic fractions.

**Fig. 5:**
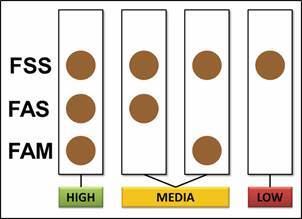
scheme proposed for a screening test using different *Angiostrongylus cantonensis* female antigens, using Dot-enzyme-linked immunosorbent assay (ELISA) technique, regarding the possibility that the patient presents neuroangiostrongyliasis.

Thus, based on better performance of FAM antigen (compared to MAM) and its higher yield in terms of protein concentration resulting from extractions, this study proposes its insertion together with FAS in algorithm for screening diagnosis of neuroangiostrongyliasis, maintaining the sensitivity of FSS and improving specificity of technique with addition of alkaline female antigens. Fig. 5 schematically demonstrates possible interpretation of results from highest to lowest probability that a patient presents neuroangiostrongyliasis in screening test.

Western blot results showed that antigenic fractions tested showed quite diverse band patterns. However, bands of 140 kDa of FSM, 24 kDa of FAS and 29 kDa of UTSM were the only ones that reacted only with serum samples from patients with neuroangiostrongyliasis, not showing cross-reactivity with other parasites. In addition, 31kDa band of FSS, also reagent to sera from patients with disease, showed cross-reactivity only with samples from patients with strongyloidiasis in this study.

## DISCUSSION

All tested antigens in this study showed bands of recognised diagnostic value between 29 kDa and 31 kDa, especially 29 kDa band, which was expressed by all adult forms of *A. cantonensis*. However, this band could not be observed in female reproductive tract. In other studies that approach these specific nematode bands, the antigens are performed with young females for 29 kDa, while tests with 31 kDa band use adult females.^18^ ^,^ ^35^ ^,^ ^36^ ^,^ ^37^

Regarding sensitivity, although all antigens had values lower than FSS, five were greater than 80%, six between 70 and 80% and others below 60%. The statistical analysis represented an opportune reinforcement for clarification of doubts in final analyses, being Kappa the test of choice. In general terms, K coefficient, developed by Jacob Cohen in 1960,^38^ is a statistical test used to measure reliability of qualitative results of analyses performed by different observers. Its objective is to provide concordance indices in addition to percentage calculations and, thus, rule out the possibility that concordance occurred by chance.^39^ In addition, the test has also been used for evaluation of alternative diagnostic tests compared to gold standards, such as analyses performed by the Brazilian Health Ministry to verify accuracy of rapid tests (IgG and IgM) for diagnosis of COVID-19, in 2020^40^ or concordance analysis of different techniques used in parasitological diagnosis addressed by Azevedo et al.^41^

In this context, it is important to emphasise that the present work did not aim to make comparisons between different tests, but to compare different antigenic fractions using one technique (Dot-ELISA). Furthermore, an antigen was not sought to replace the one already used in diagnosis, but we assessed this association could improve the infection diagnosis. Thus, high or very low agreement ratings would not meet this objective. Therefore, when Kappa results were crossed with sensitivity, specificity, PPV, NPV and accuracy results it was possible to highlight two antigens that showed reasonable agreement in the K test and expressive values in other tests: FAM and MAM, especially regarding their specificity, 91.21% and 89.01% respectively. These results contrast with 70.33% of specificity of FSS fraction, suggesting a gain of 20.88% in case of FAM and 18.68% in case of MAM. On the other hand, it is observed that sensitivity of these two fractions is 35.29% each. In this perspective, FAS fraction also stands out, showing moderate agreement with FSS and specificity of 84.62% (14.29% higher than FSS) and sensitivity of 76.47% (41.18% higher than MAM and FAM). Nevertheless, in order to improve diagnosis, they should be used in parallel with FSS, due to its high sensitivity.

Likewise, membrane saline antigenic fraction extracted from female reproductive tract (UTSM) also deserves attention, since it presented a specificity of 90.91% and an accuracy of 80.72%, although it was not possible to perform K test. These high values may be justifiable as antigen coincides with regions that showed immunogenic reactions by indirect immunofluorescence assay. Therefore, this antigen might be a strong candidate for further studies of protein sequencing, but not intended for use in diagnosis, due to difficulty in the process of obtaining female organs and low yield of antigens obtained, as mentioned previously.

The fact that antigens with potential for diagnostic use, FAM and MAM, are of membrane and in alkaline versions is not surprising, since different methods of antigenic preparations allow the obtainment of protein fractions with different characteristics. Usually, membrane antigens are those that have been lost in the process of soluble extractions, remaining concentrated in pellet as waste. In this way, the use of detergent and consequent breakdown of lipid molecules, results in proteins that did not solubilise in the previous process. For example, when comparing FSS and FSM, it is possible to observe that agreement between results is very low (K = 0.001), despite the fact that they did not present expressive variations in sensitivity and specificity. Likewise, antigenic variability can also be observed in alkaline extractions, because solubilisation and separation of polysaccharides from cell walls occur in presence of NaOH, as also observed in FAS, another antigen that presented satisfactory results in this work.

Thus, based on the higher performance of FAM antigen (compared to MAM) and its higher yield in terms of protein concentration resulting from extractions, this study proposes its inclusion together with FAS in algorithm for screening diagnosis of neuroangiostrongyliasis, maintaining sensitivity of FSS and improving the specificity of Dot-ELISA. Even if a confirmatory test is still needed, surveillance actions can be proposed for environmental analyses before Western blot results. In addition, Dot-ELISA represents a cheap and easy-to-perform alternative for screening diagnosis.

Use of 29 kDa and/or 31 kDa bands as markers in diagnostic tests for Western blot techniques has been described in several regions of the world, including non-endemic areas of the disease, as first autochthonous case occurring in France,^42^ imported cases in Germany^43^ and others registered in Brazil.^44^ Furthermore, same band may represent recognition of more than one protein, as pointed out by Morassutti et al.^37^ ^,^ ^45^ for proteins 14-3-3, Lec5 and ES7 of 31 kDa band, with use of bi-dimensional electrophoresis, pointing future perspectives for approaches to neuroangiostrongyliasis laboratory diagnosis.

As for confirmatory test (Western blot), due to difficulties in obtaining a sufficient amount of female reproductive apparatus for production of UTSM fraction for diagnosis, results of this study suggest the evaluation of use of MSM, which presented reactions in band of 31 kDa, already established in scientific literature, without crossing with other helminthic antigens. Therefore, it will be necessary to assess its efficiency with a larger sample of sera and also carry out tests with CSF samples. In addition, other bands with same performance, not yet studied, can serve as a starting point for new approaches in this area.

Thus, according to results of this study, all analysed fractions showed reactivity with serum samples from patients with neuroangiostrongyliasis. However, extracts from adult worms seem to be more appropriate for use in Dot-ELISA as a screening test, especially FAM and FAS fractions together with FSS, improving the technique specificity. We also propose introduction of MSM extract for refinement of Western blot in infection diagnosis.

In addition to these contributions, the present study brings a simple approach for purifying L1 larvae from rodent feces using semi-hydrolysed collagen, as well as a technique for removing the reproductive tract from adult females of *A. cantonensis* for antigenic research.

*Study limitations* - This study was carried out with a limited sera number since neuroangiostrongyliasis is a poorly-known disease worldwide and, consequently, our laboratory received, as of now, few cases for diagnosis. This problem could be minimised by capturing unclarified cases of eosinophilic meningitis, with epidemiological surveillance support and/or carrying out work in association with international institutions that also receive samples for this diagnosis.

Unfortunately, only one-dimensional electrophoresis was performed in this study due to lack of resources. However, this study opens up opportunities to investigate antigens using two-dimensional electrophoresis, proteomic and recombinant antigen analyses. In addition, results obtained can contribute to the improvement of laboratorial diagnosis and a systematic way, creating an algorithm with methodologies, from start of symptoms and the different infection stage.
